# Germania

**Published:** 1890-01

**Authors:** 


**Germania.** A Fortnightly Journal for the Study of the German Language and Literature, Manchester, N. H.

Any educational journal which facilitates the study of a language so important as German, is a welcome addition to the text-books upon this subject. As said in the prospectus, the aim of Germania is two-fold: (1) It will teach the language; special attention is given to the different grades of students, beginners, intermediate, and advanced. Great care and attention is given to orthoepy and orthography, to synonyms, to grammar, etc., and a special column is devoted to each of them. (2) It will try to acquaint readers with the best of German Literature. It always contains a continued novel from the pen of the best German writers, with translations of difficult words and phrases. It gives selections from the great poets, biographies, National ballads, etc. It contains a special column of books and lectures for the assistance of its readers. It answers also all questions concerning literature, grammar. pronunciation, etc. It is, indeed, a valuable journal, containing many interesting bits of information, historical and other. We hope it may receive the liberal support that it deserves. J. P.

